# Resection of iliac artery to sigmoid colon fistula in patient with prior bowel resection and endovascular aortic repair with hypogastric coiling

**DOI:** 10.1016/j.jvscit.2025.101801

**Published:** 2025-04-07

**Authors:** Michael Chaney, Nicholas Stevens, Samuel Coster, Matthew Welter, David Minnick, Saad Shebrain

**Affiliations:** a Department of Surgery, Western Michigan University Homer Stryker MD School of Medicine, Kalamazoo, MI; b Division of General Surgery, Bronson Methodist Hospital, Kalamazoo, MI

**Keywords:** Arterial-enteric fistula, Colectomy, Endovascular aortic repair, Extra-anatomic bypass, Iliac aneurysm

## Abstract

Expedient recognition of arterial-enteric fistulas is important in optimizing patient outcomes. The most commonly described aortoenteric fistula is between the abdominal aorta and third portion of the duodenum that overlies it. This has historically been reported as a rare complication of open aortic aneurysm repair but also has been seen in endovascular repairs. Herein described is a case of a 77-year-old male with history of endovascular aortic repair and left hypogastric coiling for aneurysm who presented with a much rarer form of fistula between the residual sigmoid colon, status-post sigmoidectomy for diverticulitis, and the left hypogastric artery.

Arterial-enteric fistulas (AEFs) are a rare, life-threatening condition where, as described in the name, a fistula develops between the arterial system and gastrointestinal tract. This is classically seen between the abdominal aorta and the third portion of the duodenum in patients who have had open repair for abdominal aortic aneurysm (AAA) but also has been reported following endovascular repairs. A myriad of other described fistulas, including aortoesophageal, mesenteric to duodenal, and gastroepiploic to duodenal, have been described in the literature.^1^, ^2^, ^3^, ^4^ AEFs are classified as primary and secondary. Primary AEF occurs when an aneurysm expands and subsequently erodes into neighboring alimentary tract anatomy. Secondary AEFs, which are more common than primary, are seen following aneurysm repair or trauma. Factors associated with poorer outcomes are time to treat, severity of hemorrhage, and gross contamination of stool.^5^

Both upfront definitive open repair and endovascular temporizing repair of the artery with staged fistula takedown with interval alimentary tract repair have been described.^6^^,^^7^ Careful consideration to patient stability, comorbidities, and anatomic location of the fistula must be made when selecting intervention modality. This report details a patient with AEF in an unusual location, between the sigmoid colon and left internal iliac (or hypogastric) artery. They were successfully treated via bowel resection with staged colostomy creation, aneurysm isolation and excision, and femoral-to-femoral bypass.

## Case report

**Fig 1:**
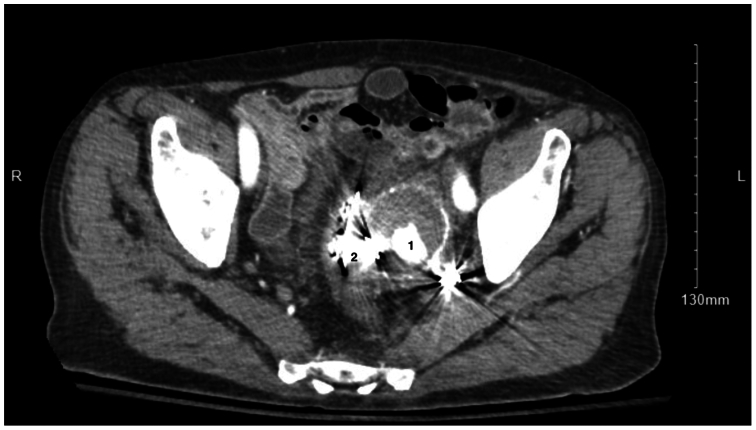
A preoperative computed tomography (CT) axial cut of the pelvis on the day of index surgery showing a patent left internal iliac artery (*1*) with surrounding aneurysm as well as contrast extravasation into the distal sigmoid colon (*2*).

**Fig 2:**
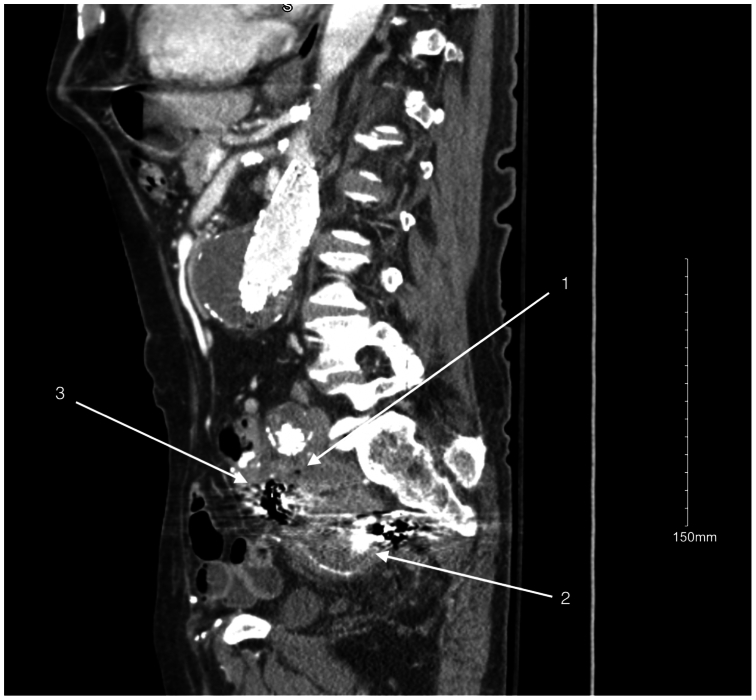
A preoperative sagittal computed tomography (CT) gas within the left hypogastric aneurysm sac (*1*), a patent left hypogastric artery (*2*), and coils that have migrated within the enlarging left hypogastric aneurysm sac (*3*).

The patient is a 77-year-old male with significant medical comorbidities including history of sigmoid diverticulitis requiring sigmoidectomy approximately 20 years prior to presentation as well as a 6.5 cm infrarenal AAA with previous endovascular aortic repair (EVAR) with bilateral iliac limb extension for common iliac aneurysms and coil embolization of a concomitant 4.5 cm left hypogastric aneurysm four years prior. The patient presented to the emergency department with gradual onset abdominal pain and hematochezia over the previous 48 hours. His initial exam demonstrated normal vital signs including being afebrile as well as a soft abdomen with no frank peritoneal signs. His laboratory tests were significant for mild anemia with hemoglobin of 11 gm/dL and a leukocytosis of 14 × 10^9^ white blood cells/L. Computed tomography (CT) imaging with intravenous contrast was notable for pelvic free air, air in the left hypogastric artery aneurysm measuring 4.3 cm in size increased from under 3 cm during initial coiling per outside records, as well as contrast extravasating into the sigmoid colon down into the rectum (Figs 1 and 2). The patient was admitted in the evening and was initially stable but then developed recurrent hematochezia and tachycardia overnight with a drop in his hemoglobin level to 6.9 g/dL prompting blood transfusion. Given the imaging findings and clinical status change, the decision was made to proceed to the operating room to address the arterial-colonic fistula emergently. First an extra-anatomic right-to-left femoral-to-femoral artery bypass was created using ringed PTFE in anticipation of left iliac artery ligation. This was due to the proximity of the gas to left common iliac artery limb extension. Next a laparotomy incision was made and encountered was what appeared to be residual diseased sigmoid colon, dissection of which was performed to allow for medial rotation. Proximal and distal arterial control of left common iliac artery was established as well as control of the hypogastric aneurysm. The fistula was now visible connecting the distal sigmoid colon and hypogastric aneurysm sac. The left common iliac artery was transected and the previously placed endograft divided. Proximally, the endograft appeared grossly normal, and therefore, the stump was oversewn without further resection of the endograft. Distally, the external iliac artery remained clamped, and the hypogastric did not appear to have back bleeding. At this point, the general surgery team completed dissecting out the residual sigmoid colon, which was diseased from diverticulitis, further mobilizing the colon proximally to the splenic flexure, identifying the old anastomosis from prior resection. During this dissection, the left ureter was identified and protected. Completion sigmoidectomy was performed by transecting the sigmoid colon proximally at the descending colon and distally at the proximal rectum using GIA staplers. The fistula between the internal iliac artery (hypogastric) aneurysm sac and sigmoid colon was noted to be approximately 8 mm in diameter. The vascular surgery team then evacuated the thrombus from the hypogastric aneurysm sac. Back bleeding was readily obtained, which was controlled with pressure. Also in the aneurysm sac, there was evidence of inflammatory process, and removal of the sac and previous coils was performed. Due to extensive scarring and concern for the deeper pelvic venous anatomy, the posterior portion of the aneurysm was left in place. The external iliac artery in the pelvis and other back bleeding vessels were ligated. Estimated blood loss was in excess of 500 mL and the patient received one unit of packed red blood cells intraoperatively. Due to the extended length of the procedure, the decision was made to leave the bowel in discontinuity and return for interval second-look laparotomy after resuscitation in the intensive care unit. Upon completion of the case, both the bypass and lower left limb had pulses on Doppler exam.

**Fig 3:**
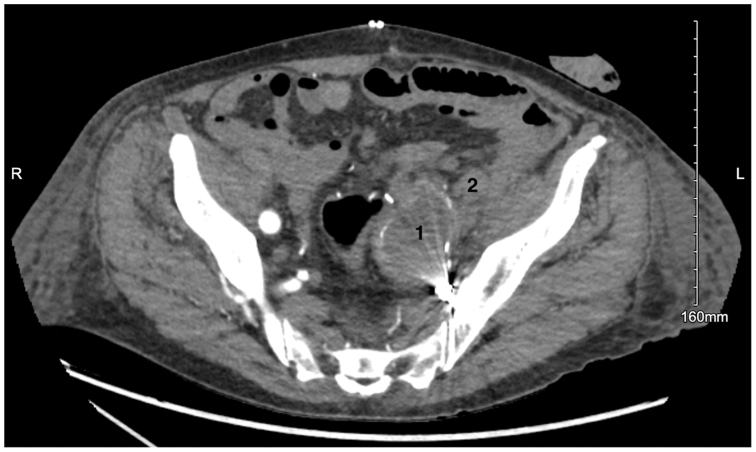
Postoperative computed tomography (CT) axial cut of the pelvis 11 days postoperatively showing the ligated left internal iliac artery (*1*) as well as the ligated left external iliac artery (*2*).

The following day abdominal washout was performed with creation of a permanent descending colostomy. After a 3-day stay in the intensive care unit, the patient was transferred to the general surgery floor. His hospital course was complicated by pneumonia requiring antibiotics, which resolved prior to discharge. Given the concern about the potential infection in the remaining aortic endograft, a multidisciplinary discussion including the infectious disease team was held. The recommendation included long-term oral antibiotics for 1 month. Upon discharge to a skilled nursing facility, the patient’s colostomy was productive, the new arterial bypass was palpably pulsatile, and he was placed on aspirin 81 mg daily. At 4-month follow-up after surgery, CT angiogram (CTA) showed both a patent EVAR and femoral-femoral bypass (Fig 3). Unfortunately, at the 7-month follow-up postoperatively, the patient presented to the emergency department with bilateral lower extremity pain, coolness, and diminished but present posterior tibial artery pulses. CTA showed a completely occluded EVAR and femoral-to-femoral bypass. The patient underwent emergent bilateral common femoral artery cutdowns. Encountered was a thrombosed bypass graft and inability to get flow from the right external iliac artery. The decision was made to perform a right axillary to bifemoral bypass with distal anastomoses on the common femoral arteries distal to the previous bypass that was left in place. Following the procedure, the patient’s pain resolved, and he was discharged from the hospital after a short stay. Upon most recent follow-up 2 years after the initial open repair, the patient continues to do well. He is asymptomatic, and his left and right ankle-brachial indices are 0.83 and 0.82, respectively. His colostomy is functioning appropriately.

The patient expressed consent to the publication of this case report.

## Discussion

Approximately 50% of patients with an identified AAA have concomitant iliac artery aneurysms, 70% of which are in the common iliac arteries.^8^^,^^9^ Hypogastric aneurysms typically can be addressed endovascularly with coiling, as this patient had received, and then coverage of the ostium with stent grafting. Although long-term follow-up data is sparse regarding success rates of coiling, it is intended to prevent back bleeding into the aneurysm sac and halt progression and eventual rupture. In our patient, the seal of the coils was not sufficient, and the sac continued to grow after the initial endovascular intervention. This, in the presence of chronic diverticular disease, allowed for fistula formation. An estimated 50% of patients over 60 years of age have diverticula present in their colons, with about 10% to 25% of those patients developing symptoms.^10^ Chronicity and severity of disease, particularly in those who perforate, are associated with increased risk for fistula formation.^11^ There is a strong association for AAAs in those with diverticular disease, and an even stronger association for AAAs in those with diverticular disease for 10 years or more.^12^

Although a staged approach to AAA, and other large vessel disease, with concomitant disease in the abdomen, has been seen to improve outcomes,^13^ the acuity of this patient’s presentation did not allow for staged intervention. A study comparing open aneurysm repair and EVAR with staged repair of other concomitant surgical pathology EVAR had a 0% mortality rate, whereas simultaneous open repair was 13.6%; this finding was significant, albeit with a small sample size.^13^ Other case reports of AEF describe both combination open and endovascular approaches^14^ as well as primary solely open repair.^15^^,^^16^ One publication described a case series of AEFs between the thoracic aorta and esophagus after thoracic EVAR. It noted that open reoperation was extremely difficult, and there was a preference towards endovascular reintervention as a means of definitive repair without tract ligation of the fistula.^16^ Although the patient in this case report did not undergo initial endovascular temporization, in hindsight, it may have been possible to temporize bleeding either via repeat coiling or embolization of the left hypogastric artery as opposed to a difficult exposure in an inflamed pelvis.

In our case, due to the connection between the enteric system and the aneurysm containing the endograft, the decision was made to remove a portion of the stent graft to avoid long-term infectious complications. Aortic and iliac endograft explantation is a morbid procedure with 30-day mortality being reported at 6.3%, with those receiving iliac limb removal having increased risk of iliac artery degeneration.^17^ Partial explantation of endograft remains a debated topic, and recent analysis has shown higher complication rates and hospital stays with partial explantation, albeit with similar short-term mortality and long-term survival.^18^ Current recommendations for AEF with explantation of contaminated endograft include long-term antibiotic coverage for enteric flora as well as bypass in an uncontaminated field, in this patient a femoral-to-femoral bypass.^19^ This has been challenged by some who have seen comparable outcomes between in situ bypass and extra-anatomic bypass.^20^

In summary, we report an uncommon scenario of a patient with an AEF arising between the sigmoid colon and a left hypogastric artery aneurysm who underwent initial open resection and extra-anatomic bypass with femoral-to-femoral bypass and interval colostomy creation. Although he did have thrombosis of the repair in under a year, ultimately his outcome was fair, and he remains ambulatory. Careful consideration of anatomic location and patient comorbidity must be taken when addressing this life-threatening condition.

## Conclusion

Here, we report a rare internal iliac artery to sigmoid colon fistula in a patient with history of both bowel resection and EVAR with hypogastric aneurysm coiling. Time to diagnosis and treatment is critical in optimizing patient outcome as delayed diagnosis and persistent hemorrhage drives mortality. Careful consideration must be made to anatomy and acuity when selecting intervention modality.

## Funding

None.

## Disclosures

None.
